# Current Perspectives on the Contralateral Patent Processus Vaginalis: What About the Other Side?

**DOI:** 10.7759/cureus.103451

**Published:** 2026-02-12

**Authors:** Dimitrios Godosis, Vasileios Mouravas, Paschalis Theotokis, Sofia Gargani, Dimitrios Sfoungaris, Maria Eleni Manthou, Soultana Meditskou, Ioannis Spyridakis

**Affiliations:** 1 Department of Pediatric Surgery, School of Medicine, Faculty of Health Sciences, Aristotle University of Thessaloniki, Thessaloniki, GRC; 2 First Department of Pediatric Surgery, School of Medicine, Faculty of Health Sciences, General Hospital of Athens "G. Gennimatas", Aristotle University of Thessaloniki, Thessaloniki, GRC; 3 Laboratory of Histology and Embryology, School of Medicine, Faculty of Health Sciences, Aristotle University of Thessaloniki, Thessaloniki, GRC; 4 First Department of Pediatric Surgery, General Hospital of Athens "G. Gennimatas", Aristotle University of Thessaloniki, Thessaloniki, GRC; 5 Second Department of Pediatric Surgery, School of Medicine, Faculty of Health Sciences, Papageorgiou General Hospital, Aristotle University of Thessaloniki, Thessaloniki, GRC

**Keywords:** contralateral patent processus vaginalis, histological characteristics, laparoscopic surgery, metachronous contralateral inguinal hernia, pediatric inguinal hernia

## Abstract

Inguinal hernia (IH) repair is frequently performed in pediatric surgery, and the condition appears more often in children born prematurely. A key clinical concern is the possibility of developing a metachronous contralateral inguinal hernia (MCIH). This review discusses potential mechanisms involved in pediatric IH formation, with particular focus on how smooth muscle differentiation and fibrotic remodeling may contribute to incomplete closure of the processus vaginalis (PV). It also considers how evolving diagnostic approaches and surgical techniques have shaped contemporary management. In addition, histopathological evaluation may provide further understanding of PV architecture, support more tailored operative decision-making, and help limit the need for subsequent procedures. Overall, the review emphasizes the importance of investigating the histological and ultrastructural features of IHs and hydroceles, and suggests that variations in smooth muscle differentiation and myofibroblast-related changes within the PV may be linked to persistence of patency and susceptibility to herniation.

## Introduction and background

Introduction

Inguinal hernia (IH) repair remains one of the most common pediatric surgeries, with an incidence in children ranging from 0.8% to 5% [1-3]. IHs are usually unilateral. The risk of developing a metachronous contralateral inguinal hernia (MCIH) is significant, affecting 7% to 15% of children with unilateral hernia [4,5]. This phenomenon is largely attributed to the unclosed remnant of the tunica vaginalis, known as patent processus vaginalis (PPV), which is an open peritoneal extension that should have closed after birth. Therefore, a contralateral patent processus vaginalis (CPPV) is a potential site for future herniation [4,5].

Premature infants are particularly affected, with rates between 16% and 25% due to a higher frequency of a patent processus vaginalis (PV) that has yet to close [6]. Notably, at birth, approximately 80% of PVs are still open, with a sharp decrease in patency by six months of age. However, not all open PVs progress to clinical hernias.

The incidence of clinical IH is significantly higher in males, with recorded ratios ranging from 3:1 to 10:1 in full-term infants. Interestingly, this gender disparity does not appear in premature infants [3,7]. Approximately 60% of clinical inguinal hernias occur on the right side, with similar frequencies in boys and girls [8,9]. This observation in boys is often attributed to the delayed descent of the right testis compared to the left, resulting in later closure of the right PV [9]. However, this theory does not explain the similar prevalence on the right side among girls [10]. Bilateral inguinal hernias occur in about 10% of cases [9,11]. Additionally, children with an initial left-sided hernia are more likely to develop a future right-sided hernia, though recent studies challenge this observation [12-15]. Around 11.5% of pediatric patients with IH have a family history of the condition [3,16]. Higher incidences have also been observed among twins, with rates of 10.6% in male twins and 4.1% in female twins [17].

Background

Research suggests that 80% to 100% of newborns are born with an open PV bilaterally, and closure generally occurs within the first six months of life [18]. After this period, PV patency rates drop, plateauing between ages three and five. Following regression, the PV remains as a thin fibrous cord that eventually disappears and becomes integrated into the external spermatic fascia [18,19]. Several pathophysiological mechanisms contribute to the closure of the PV. Hormonal regulation, cellular events such as apoptosis, and processes of tissue remodeling all play essential roles. Pathologists’ reports indicate that disturbances in the epithelial-mesenchymal transition may underlie failure of normal closure of the PV, resulting in its persistent patency and consequently increasing the risk of inguinal hernia [20,21].

Fibrosis and tissue contraction contribute to the closure of the PV, mediated primarily by smooth muscle cells and/or myofibroblasts. Immunohistochemical studies utilizing markers such as the α-smooth muscle actin (α-SMA) and vimentin have shown increased fibroblast activity within PVs, suggesting that these pathways may contribute to the pathogenesis of inguinal hernia [2]. Consistent with this hypothesis, the findings of Tanyel et al. suggest that the propensity for hydrocele or hernia is influenced by the density of these muscle cells, as well as by the involvement of the sympathetic nervous system and calcium ion channels within the muscle cells [22,23].

Supporting this theory, Mouravas et al. have correlated the clinical manifestation of the PV with the degree of smooth muscle cell differentiation. In pediatric cases of indirect inguinal hernia or hydrocele, the walls of the PV sac show smooth muscle fibers that differ histologically from those in closed PVs, where smooth muscle fibers are absent [24]. Additionally, the distribution and expression of immunohistochemical markers for differentiated smooth muscle cells correspond to the type of PV abnormality [24,25]. Immature smooth muscle fibers within hydrocele sacs express vimentin, indicative of undifferentiated cells undergoing programmed cell death, whereas mature smooth muscle markers such as h-caldesmon, desmin, and α-SMA are more frequently detected in both hydrocele and hernia sacs. These findings suggest that hydrocele sacs contain less differentiated fibers compared to the hernia sacs [26].

This review integrates clinical, surgical, and histopathological data to clarify the role of contralateral exploration in pediatric unilateral inguinal hernia and its impact on CPPV detection, MCIH prevention, and postoperative outcomes.

## Review

Methods

We reviewed the available literature to evaluate approaches for detecting CPPV and preventing MCIH in children. PubMed and Scopus were searched without date restrictions for studies comparing laparoscopic and open approaches for contralateral exploration. Studies were included if they met the following criteria: (1) pediatric patients with unilateral inguinal hernia and no clinical evidence of contralateral hernia; (2) comparative data on laparoscopic and open exploration; and (3) reported outcomes on CPPV incidence, MCIH occurrence, and postoperative complications. Clinical, surgical, and histopathological data were extracted where available to integrate outcome analysis with structural and ultrastructural findings.

Clinical approaches

Epidemiology and Risk Stratification

IHs are one of the most common surgical conditions in the pediatric population, with a prevalence up to 5% for infants and more than 30% in preterm infants. Therefore, the risk of developing MCIH is highest in children under six months [1,2,27-29]. An interesting 10-year review found that 44% of children younger than one year had a CPPV, in comparison with a 17% rate of incidence in older kids [30]. These data further support the significance of early contralateral assessment, especially in infants, as spontaneous closure tends to occur more frequently beyond infancy.

Operative Strategies: Contralateral Exploration and Repair

It seems that the benefits of contralateral exploration in reducing MCIH risk are rather significant. There are several laparoscopic techniques that gained popularity over the last two decades due to reduced postoperative pain, shorter recovery, and reduced recurrences [31,32]. Laparoscopic surgery provides a clear view of the abdominal cavity, allowing the assessment and management of both the hernia and the contralateral side. The most common process is with a camera that is introduced through an umbilical port [29,33]. Additional ports facilitate the surgical procedure [33,34]. This approach allows surgeons to inspect both inguinal rings in real-time, offering a significant advantage over open methods. More specifically, under general anesthesia and using a three-port laparoscopic technique [35], a peritoneal incision is made to access the hernia sac, and the closure of the internal ring ensures that the hernia sac is contained within the abdominal cavity. It is crucial for the contralateral inguinal region to be inspected to identify a patent PV [33,35].

A different laparoscopic method is the transinguinal laparoscopic exploration (TILE) that allows for detailed inspection and repair of the contralateral PV and minimizes future surgeries. More specifically, TILE has been shown to reduce MCIH incidence from 13.6% in non-explored cases to 4.3% when the contralateral side is evaluated [1,27].

A meta-analysis by Dreuning et al. reported a sensitivity of 93% and specificity of 88% when using ultrasonography for CPPV detection [29]. These findings support the potential use of ultrasonography as a preoperative screening tool. However, authors observed a tendency to overestimate MCIH prevalence. Therefore, there is a need for standardized diagnostic parameters to avoid unnecessary interventions [29].

Laparoscopic repair techniques, particularly laparoscopic percutaneous extraperitoneal closure (LPEC), have shown advantages over open surgery repair in pediatric inguinal hernia cases. A review compares laparoscopic and open techniques and reports that laparoscopic herniorrhaphy is associated with shorter operative times (OTs) for bilateral repairs and lower postoperative complication rates. These complications include infection and recurrence, which are more common with open surgery. On the other hand, they report that laparoscopic approaches reduce the incidence of MCIH, suggesting that these techniques may offer long-term advantages for high-risk pediatric populations [34].

Similarly, Kurobe et al. demonstrated that LPEC is associated with a significantly lower incidence of MCIH compared to open surgery [36]. These findings suggest that laparoscopic techniques are more efficient in addressing CPPV during the initial procedure and may prevent the recurrence of herniation postoperatively [36]. Nevertheless, LPEC does not come without potential complications. More specifically, LPEC was more associated with the umbilical port site (UPS) and related complications [36]. Interestingly, Zhao et al. indicate that laparoscopic techniques generally have shorter OTs for bilateral repairs and a reduction in postoperative pain scores compared to open surgery (Table 1) [34].

**Table 1:** Comparison of laparoscopic and open surgical techniques for pediatric inguinal hernia repair. MCIH: metachronous contralateral inguinal hernia; UPS: umbilical port site; SSI: surgical site infection.

Outcomes	Laparoscopic techniques	Open repair
Incidence of MCIH	0% (Kurobe et al., 2021) [36]	4.1% (Kurobe et al., 2021) [36]
Recurrence rate	0.3% (Zhao et al., 2021) [34]	0.4% (Zhao et al., 2021) [34]
UPS-related complications	Higher (e.g., superficial SSI) [31,33,34]	Lower
Operative time (bilateral)	Shorter (Zhao et al., 2021) [34]	Longer
Postoperative pain	Lower (Zhao et al., 2021) [34]	Higher

Diagnostic Evaluation: Ultrasonography

A meta-analysis by Dreuning et al. reported a sensitivity of 93% and specificity of 88% when using ultrasonography for CPPV detection [29]. These findings support the potential use of ultrasonography as a preoperative screening tool. However, authors observed a tendency to overestimate MCIH prevalence. Therefore, there is a need for standardized diagnostic parameters to avoid unnecessary interventions [29].

Structural and Ultrastructural Studies on PV Pathology

Electron microscopy offers valuable insights into PV-related conditions by enabling detailed analysis of the ultrastructural characteristics of the relevant anatomical structures. More specifically, ultrastructural studies have shown that the hernia sacs in the pediatric population with clinical indirect inguinal hernia or hydrocele features present myofibroblasts within the smooth muscle fibers of the sac. This observation reflects an apoptotic process in smooth muscle fibers, with dedifferentiation occurring at an earlier developmental stage, indicated by the presence of myofibroblasts [26].

It is well-established that smooth muscle cell proliferation results from dedifferentiation into myofibroblasts, which exhibit characteristics of both fibroblasts and smooth muscle cells [37]. When dedifferentiated cells undergo sufficient mitotic activity, they eventually cease proliferation and redifferentiate [38,39]. Smooth muscle cells uniquely transit through a cycle of differentiation, dedifferentiation, and redifferentiation.

In examining hernia and hydrocele sacs in children, myofibroblasts are more concentrated in clinical hernia sacs than in hydrocele sacs. Given that many pediatric hydroceles resolve spontaneously over time, it is hypothesized that the presence of more undifferentiated smooth muscle fibers in hydroceles may facilitate PV closure. In contrast, higher concentrations of myofibroblasts in hernias suggest a difficulty with spontaneous PV closure [24].

Histological and immunohistochemical studies can offer valuable insights into PV-related pathophysiology in hernia cases. Furthermore, ultrastructural studies revealed that immature smooth muscle fibers are more prevalent in hydrocele sacs, suggesting a natural propensity for closure. On the other hand, higher concentrations of differentiated fibers in hernia sacs present a lower risk of recurrence [23,24,29].

The clinical implications of these findings are significant for the management of pediatric inguinal hernias and hydroceles. By identifying specific histological and ultrastructural features of the PV, surgeons can better predict the likelihood of hernia development or spontaneous resolution in pediatric patients. This approach could potentially reduce the rate of MCIH, thereby decreasing the need for secondary surgical interventions.

Where do we stand now?

Surgeons are skeptical regarding contralateral exploration during unilateral hernia repair. While many agree that it may prevent MCIH, others believe that it may be unnecessary and adds risk without benefits​ (Table 2) [5,28].

**Table 2:** Key findings and recommendations. MCIH: metachronous contralateral inguinal hernia; CPPV: contralateral patent processus vaginalis; PV: processus vaginalis.

Aspect	Summary
Key findings	Laparoscopic exploration reduces MCIH risk and allows simultaneous management of CPPV. Histopathology shows smooth muscle differentiation, and myofibroblast presence correlates with PV closure potential. Ultrasonography shows high sensitivity for CPPV but requires standardization.
Recommendations	Consider contralateral exploration in high-risk infants (<6 months). Employ preoperative ultrasonography where appropriate. Consider selective histopathological analysis in recurrent cases.
Clinical implications	Individualized surgical decisions may reduce unnecessary reoperations, optimize outcomes, and minimize anesthesia exposure in high-risk populations.
Future directions	Correlate histopathological findings with long-term clinical outcomes. Investigate molecular pathways of PV closure for targeted therapies. Establish guidelines integrating laparoscopic exploration, ultrasonography, and histopathological data for personalized management.

Therefore, the decision to explore may need to be individualized based on patient age and other risk factors, such as initial hernia side and PV characteristics. Dreuning et al. attempt to explore the professionals' perspectives on routine contralateral exploration during unilateral hernia repair [27,40]. The study agrees on the surgical risks and the unclear benefits. However, it was acknowledged that there is a beneficial role in this approach for high-risk infants in terms of reducing anesthesia and surgery [40,41]. Moreover, infants have shown the highest susceptibility to MCIH due to delayed or incomplete closure of the PV, and therefore, contralateral exploration seems to have a strong indication in this population [36].

Additionally, the increasing use of preoperative ultrasonography has shown promise in detecting CPPV with high diagnostic accuracy. As highlighted in Dreuning et al., ultrasonography offers a non-invasive approach to detect CPPV, achieving sensitivities as high as 93% [29]. However, standardizing diagnostic criteria remains crucial, as variability across studies may lead to overestimations of MCIH risk (Table 2) [29].

Recent advances in laparoscopy

Laparoscopy has transformed contralateral exploration, offering a minimally invasive method with reduced postoperative recovery, minimal scarring, and precise visualization of the internal ring [2,21,33,35,42].

A large retrospective study reports that the use of a double stitch technique, either a Z-stitch or an additional purse-string suture, is associated with a lower recurrence rate of hernias [43].

TILE is a particularly effective technique, allowing direct visualization and, when necessary, repair of the contralateral PV without extensive surgical exposure. This approach has been linked to lower rates of postoperative complications, positioning it as a preferred option for pediatric patients requiring hernia repair [1].

The LPEC technique, as evaluated by Kurobe et al. (2021), has shown a significant reduction in the incidence of MCIH compared to open repair. The absence of MCIH in patients undergoing LPEC indicates the procedure’s preventive potential against secondary hernias. Despite these advantages, LPEC may present specific challenges, such as UPS complications, underscoring the importance of technical precision in preventing postoperative sequelae [36].

A modified single-needle LPEC technique is described by Zeng et al. [44]. Instead of ports, the procedure used a specialized needle for a purse-string suture around the hernia. This method lowers the risk of injuries [44].

Eurlings et al. explored the benefits of a robotic-assisted surgical platform and concluded that the method is safe and feasible; however, until now, with no significant benefits in comparison with the classic laparoscopic techniques [45].

Regarding the recent advantages in laparoscopy and the comparison to open surgery, Carter et al. report that in terms of recurrences, laparoscopic techniques (usual and modified) do not differ significantly from open repair [46]. In contrary, postoperative pain and the recovery time seem to be the benefits from every laparoscopic technique in comparison to open surgery [46]. Similarly, Kılıç et al. compared laparoscopic percutaneous internal ring suturing with open surgery and presented a mean unilateral OT approximately five minutes shorter than open surgery [47]. Also, the laparoscopic technique allowed the evaluation of the contralateral inguinal ring [47]. Despite the benefits of laparoscopy, there are challenges regarding the UPS, and of course, let us not forget the learning curve for laparoscopic techniques. Moreover, there is a significant cost of equipment [48].

Histopathological insights

A Canadian survey addressed the practice of routine pathological examination of hernia sacs in pediatric patients. Even though the majority of surgeons did not perform these analyses, pathologists identified significant abnormalities in 71% of cases. These findings suggest that histopathological evaluation may provide beneficial information, particularly in recurrent hernias, by revealing underlying factors that contribute to hernia development or recurrence [24,25,30].

Integrative perspective and clinical implications

The persistence of a PPV represents a necessary but not sufficient condition for the development of pediatric IH. Although newborns, especially preterm, exhibit bilateral patency at birth, spontaneous closure occurs in most cases during early infancy, while only a subset progress to clinical IHs [18].

Accumulating histopathological and ultrastructural evidence suggests that closure of the PV is an active, biologically regulated process, rather than a passive postnatal event. In particular, smooth muscle cells and myofibroblasts appear to play a central role in mediating fibrosis and tissue contraction during PV obliteration [2]. Importantly, the structural composition of the PV differs between conditions that tend to resolve spontaneously and those that progress to herniation. Hydrocele sacs exhibit immature or partially differentiated smooth muscle fibers, whereas IH sacs are characterized by differentiated smooth muscle fibers and myofibroblast-rich architecture [22-26].

From a clinical standpoint, these observations provide a unifying biological framework for understanding why CPPV does not uniformly translate into MCIH. They also underscore the limitations of relying solely on anatomical detection of patency when estimating future IH risk, as the likelihood of progression appears to be influenced by underlying tissue differentiation and remodeling processes that are not readily captured by clinical examination or imaging modalities alone [18,24].

By integrating developmental biology with clinical observation, this perspective supports the concept that pediatric IH represents a spectrum of PV maturation rather than a binary open-closed state. Appreciating this continuum helps reconcile discrepancies in reported incidence rates and clinical outcomes, and provides a biological rationale for individualized rather than uniform management strategies in pediatric IH repair [18,22-26].

What does the future hold?

A comprehensive understanding of the mechanisms underlying PV closure in pediatric patients is essential for advancing surgical techniques. For instance, TILE is rather promising in reducing the MCIH incidence of the contralateral PV during initial hernia repair [48]. Future studies should focus on the investigation of the mechanisms of PV closure and correlate histological findings with the long-term outcomes. The main goal is to be able to establish new guidelines, focusing on specific treatment strategies individually for each case. It seems that personalized surgical decisions based on molecular profiling are the future, to be able to cope with the discrepancies in recurrence rates and outcomes across studies. This approach will also reduce exposure to unnecessary surgical risks (Table 2).

Current evidence suggests that infants under six months with unilateral hernia may benefit from contralateral exploration due to their elevated risk of MCIH [30]. Establishing standardized criteria for CPPV detection, including the use of preoperative ultrasonography, could improve diagnostic consistency and reduce unnecessary explorations [27]. Furthermore, selective histopathological evaluation of recurrent cases may provide insights into the causes of PV persistence and help identify factors associated with hernia recurrence [30]. By integrating clinical, surgical, and molecular insights, the next generation of management strategies can move toward precision-guided, risk-adapted interventions that minimize morbidity and optimize long-term outcomes for each child.

Limitations

This review has several limitations. First, although the search strategy included PubMed and Scopus, the evidence base meeting our predefined comparative criteria was limited, and Table 1 therefore includes only comparative studies. Second, heterogeneity in patient populations (age, prematurity status, index hernia side), definitions of CPPV/MCIH, ultrasonography protocols, and operative techniques limits direct comparability and precludes formal pooled estimates within this narrative synthesis. Third, we did not perform a formal risk-of-bias assessment, and publication and reporting bias cannot be excluded. Finally, histopathological and ultrastructural observations were variably reported and were not consistently linked to long-term clinical endpoints, limiting the strength of mechanistic inferences.

## Conclusions

This study highlights the importance of CPPV exploration and the benefits of understanding the histological and ultrastructural properties of the PPV in managing pediatric patients with inguinal hernias and hydroceles. Some of the current studies suggest that the exploration of the contralateral inguinal ring for CPPV during unilateral hernia repair is important to prevent the development of MCIH. The findings support the hypothesis that the balance of smooth muscle differentiation and myofibroblast presence within the PV in clinical inguinal hernias influences its patency and the subsequent type of herniation. Further, the identification of immunohistochemical markers, such as α-smooth muscle actin and vimentin, provides insight into cellular behaviors associated with PPV closure or persistence. Future studies should focus on high-risk patients who would benefit most from contralateral exploration.
